# Capacity of blood plasma is higher in birds breeding in radioactively contaminated areas

**DOI:** 10.1371/journal.pone.0179209

**Published:** 2017-06-29

**Authors:** Magdalena Ruiz-Rodríguez, Anders P. Møller, Timothy A. Mousseau, Juan J. Soler

**Affiliations:** 1 Departamento de Ecología Funcional y Evolutiva, Estación Experimental de Zonas Áridas, CSIC, La Cañada de San Urbano, Almería, Spain; 2 Ecologie Systématique Evolution, CNRS, Université Paris-Sud, AgroParisTech, Université Paris-Saclay, Orsay, France; 3 Department of Biological Sciences, University of South Carolina, Columbia, South Carolina, United States of America; Universita degli Studi di Milano-Bicocca, ITALY

## Abstract

**Background:**

Environmental pollution in general, and radioactive contamination in particular, may deeply affect host-parasite relationships and their consequences for the evolution of organisms. The nuclear accident that occurred more than 30 years ago in Chernobyl resulted in significant changes in diversity and richness of microbial communities that could influence characteristics of animal-bacteria interactions, including host immune responses and competitive interference by bacteria. Given the high mortality rate of birds breeding in radioactively contaminated zones, those with stronger defences against infections should experience significant fitness advantages.

**Methodology/Principal Findings:**

Here we characterized antimicrobial capacity of barn swallows (*Hirundo rustica*) from different Ukrainian populations (subject to a gradient of ionizing radiation) against 12 bacterial species. We also quantified constitutive innate immunity, which is the non-specific first barrier of protection of hosts against microbial parasites. We found a positive association between specific antimicrobial capacity of individual hosts and radiation levels in breeding habitats even after controlling for other confounding variables such as sex and age. However, no significant relationship was found between immunocompetence (non-specific response) and background radiation.

**Conclusions/Significance:**

These results suggest that radiation selects for broad antimicrobial spectra of barn swallows, although not for all bacterial strains. We discuss these results in the framework of host-parasite evolution under extreme environmental conditions.

## Introduction

Host-parasite interactions are one of the main forces driving evolution [1]. Hosts are exposed to a plethora of parasites with variable fitness consequences. Parasite-host associations may depend on abiotic factors [2], so drastic changes in environmental conditions, such as contamination, could alter these relationships [3]. Environmental pollution has been shown to influence not only the biology of parasites and hosts separately, but also the investment in defences of hosts against parasites (reviewed in [3]).

Since 1945, nuclear weapons testing and accidents at nuclear facilities have increased ambient levels of ionizing radiation in many parts of the world and this can be considered an extreme environmental perturbation resulting from human activities that has drastically affected many natural populations [4]. The worst nuclear accident to date took place in 1986 at the Chernobyl Nuclear Power Plant in Ukraine, during which huge amounts of radioactive isotopes were released into the environment causing elevated mutation rates in organisms [5] and altered ecosystem functioning [6]. Due to the proliferation of nuclear power plants in the 1960’s and 1970’s, and many new plants under construction around the world, there is an increasing interest in studying effects of radiation on wildlife [7, 8, 9]. Chernobyl provides a unique opportunity for studying the ecological and evolutionary consequences of ionizing radiation on living organisms more than 30 years following the accident, allowing for assessment of adaptations to this new environment.

Since host-parasite dynamics are affected by a variety of biotic and abiotic factors, including physiological characteristics of counterparts, dynamics of host-parasite interactions are even more complex in radioactively contaminated sites, because of uncertainty of the effects on different parasites and hosts [3]. On one hand, anthropogenic radiation has a negative effect on abundance and diversity of microorganisms [10, 11]. However, due to short generation times, some microorganisms can accumulate beneficial (and detrimental) mutations that allow them to rapidly adapt to changes in their environment [12, 13], resulting in increases in abundance and distribution of resistant species within radioactively contaminated areas [14, 15]. Therefore, drastic environmental modifications after a nuclear accident may lead animals to confront a severely changed microbial community that, mainly because of the direct or indirect negative effects of radiation on host phenotype [16], may become more virulent for hosts [17], and increase the probability of successful infection. For instance, a significant percentage of atypical mycobacteria occurred in cattle from polluted areas of Ukraine following the Chernobyl disaster [18]. Therefore, it is likely that host-parasites dynamics vary in areas differing in radioactive contamination.

Animals living in radioactively contaminated areas experience depressed immunity, mainly due to profound damage to their immune system, and this may result in faster disease progression [3, 16]. Supporting this hypothesis, birds living in Chernobyl have demonstrated low levels of immune function [19], depressed levels of several types of leukocytes and immunoglobulins, and smaller spleens [5, 19]. These effects could, among others, be due to the high level of DNA damage experienced by birds living in contaminated areas [20, 21] that would affect the dynamics and the outcomes of the interaction between hosts and parasitic microorganisms.

In the present study, we characterized the immunological capacity to fight against species-specific and general bacterial infections in barn swallows *Hirundo rustica* breeding along a gradient of radioactive contamination in Ukraine (including populations from Chernobyl). First, we directly measured the capacity of blood plasma from barn swallows to inhibit bacterial growth by means of antagonistic plates in which the plasma is confronted with different standard bacterial strains. Second, we measured the constitutive innate immunity, which provides the first-line of protection against invading microbes. Specifically, we studied two humoral components: natural antibodies and complements [22, 23, 24, 25] (Carroll & Prodeus, 1998; Thorton et al. 1994; Ochsenbein & Zinklernagel, 2000; Matson et al., 2005).

Given the scenario described above, we might expect a lower defence capacity against microorganisms in barn swallows living in more contaminated areas. Alternatively, selective pressures exerted by evolving, more complex communities of microorganisms, together with high mortality rates in barn swallow hosts in Chernobyl [26], may have accelerated the process of adaptation in these bird populations. To date, there are very few examples of animals adapting to ionizing radiation in Chernobyl [27] but among these a higher level of resistance of feathers to degradation by keratinolytic bacteria was found in barn swallows living in more contaminated areas around Chernobyl [28]. In light of this prior finding, we predict that barn swallows in Chernobyl will be more resistant to bacterial infections than control populations given more than 30 years of selection following the accident.

## Methods

### Sampling procedure

**Fig 1:**
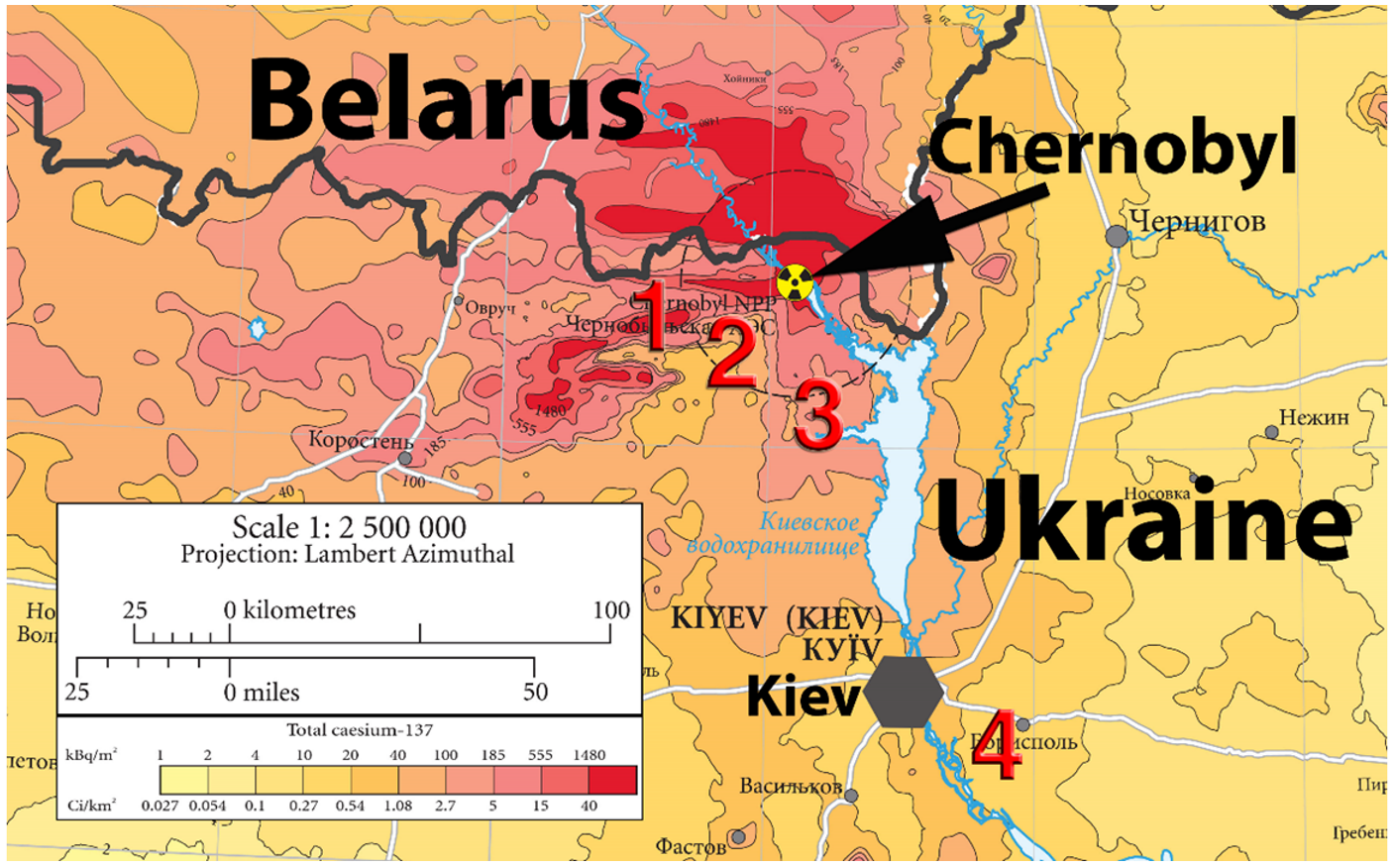
Map with all the study populations: (1) Vesniane, (2) Rudnia, (3) Dytiatki, and (4) Voronkov. The arrow denotes the nuclear power plant. Adapted from [29].

Field work was performed in May-June 2015 in Ukraine. We sampled two populations inside the Chernobyl Exclusion Zone (Rudnia (51.17.308N-29.46.536E) and Vesniane (51.17.845N-30.38.451E), < 30 km from the nuclear power plant), whose ambient background radiation levels were 1.5–2 μSv/h; another population outside but close to the exclusion zone (Dytiatki (51.06.741N-30.10.340E), < 1 km), with background radiation levels of 0.2–0.5 μSv/h; and a “clean” population located at > 100 km of the exclusion zone (Voronkov (50.22.235N-30.89.967E), 0.02–0.12 μSv/h) (see Fig 1). The external radiation levels were measured at ground level in each population (i.e., in each farm) using a hand-held dosimeter (Model: Inspector, International Medcom, Inc., Sebastopol, CA, USA), which was deposited in the ground until the measure stabilized. Values were highly consistent within sites among years. For more details on study areas, see [11].

Barn swallows were captured by placing mist nets at the entrance of farm buildings where they breed. The sex and age of each individual was determined because this could affect immunocompetence, which is defined as the ability to raise an efficient defense against parasites [30]. Since barn swallows show very low dispersal rates once they have chosen a breeding territory in their first year of life [31], and Ukrainian populations have been monitored since 2000, the age of individual barn swallows in our study sites can be accurately estimated by considering unbanded birds at first capture as being yearlings (see procedure in [32]). Blood samples were taken by puncturing the brachial vein and collected in heparinized capillary tubes. The blood was deposited in a MiniCollect tube (0.8 mL, LH Lithium Heparin Sep.), and then briefly centrifuged (12,000 g, 5 min) to separate the plasma fraction. Plasma was stored at -20°C until lab analyses could be performed during the following two months. Sampling effort (i.e. time devoted to capture of birds) was similar in all populations. We captured in total 85 individuals, 41 females and 44 males; 15 individuals were captured in Rudnia (6 females and 9 males), Vesniane (6 females and 9 males) and Dytiatki (7 females and 8 males), and 40 individuals (22 females and 18 males) in Voronkov.

Birds were captured and released about 10 min later. All individuals flew after release and appeared to be unaffected by handling. No birds were damaged or died as a consequence of manipulation. Permit for capturing, handling and blood withdrawal of the birds was given by the Chernobyl Exclusion Zone Authority. No anesthesia, euthanasia, or any kind of animal sacrifice was performed.

### Antimicrobial activity of plasma

Antimicrobial tests were performed against twelve indicator bacteria (listed in Table 1) from a broad range of taxonomic groups. Antagonistic plates were prepared as follows: 15 mL of a culture medium previously prepared and sterilized (1.8% of brain–heart infusion (BHI) and 0.8% agar in 0.1 M pH 7 phosphate buffer) was melted and then maintained at 50°C for 10 min. Then, 100 μL of a 12-h culture of each indicator bacteria (Table 1) was added to the medium, vigorously vortexed and spread onto a Petri dish. After solidifying about 30 min later, 2 mL of each plasma sample was placed on the plates and later incubated for 12 h at 28°C. After incubation, plates were checked for inhibition halos, that is, transparent zones around the plasma in which the growth of the indicator bacterium was inhibited. Halos were measured (in mm) from the limit of the plasma drop to the end of the halo (i.e., where the indicator bacteria growth begins). Antagonistic tests against each species were made to all the samples in the same randomly selected order (listed in Table 1). For some of the samples, there was insufficient plasma for testing all indicator bacteria (N of each assay is given in Table 1).

**Table 1:** Results of multiple regression analyses in which the radiation level was the independent variable, and inhibition capacity (i.e., halo size) was the dependent variable. *Q* is the statistical probability after adjustment for number of tests. The asterisk (*) denote significant values.

Indicator bacteria	Beta (SE)	*F*	d.f.	*R*^2^	*P*	*Q*	*N*
*Enteroccus faecium*	0.39 (0.10)	15.02	1, 83	0.14	0.0002	0.0004*	85
*Listeria monocytogenes*	0.19 (0.10)	3.39	1, 82	0.03	0.06	0.06	84
*Listeria inocua*	-0.32 (0.10)	9.63	1, 82	0.09	0.0026	0.003*	84
*Lactobacillus paracasei*	-0.49 (0.09)	25.82	1, 82	0.23	<0.0001	0.0002*	84
*Lactococcus lactis*	0.60 (0.09)	42.94	1, 77	0.35	<0.0001	0.0002*	79
*Lactobacillus plantarum*	0.22 (0.11)	3.76	1, 74	0.03	0.05	0.06	76
*Enterococcus faecalis*	0.61 (0.09)	43.90	1, 72	0.37	<0.0001	0.0002*	74
*Bacillus thuringiensis*	0.61 (0.09)	41.54	1, 69	0.36	<0.0001	0.0002*	71
*Bacillus licheniformis*	0.40 (0.11)	13.55	1, 68	0.15	0.0004	0.0006*	70
*Proteus* sp.	0.37 (0.11)	10.86	1, 68	0.12	0.001	0.0015*	70
*Bacillus megaterium*	-0.17 (0.12)	2.11	1, 65	0.01	0.15	0.15	67
*Staphylococcus aureus*	0.65 (0.09)	45.31	1, 62	0.41	<0.0001	0.0002*	64

### Immune assays

We estimated the immune response mediated by natural antibodies and complement by following the procedure described by Matson et al. (2005). Briefly, 50 μL of plasma were serially diluted in sodium phosphate buffer (PBS) in two consecutive polystyrene 96-well assay plates where 25 μL of 1% rabbit blood cell suspension (Hemostat laboratories, Dixon, CA 95620, USA) in PBS was added. Quantification of lysis and agglutination titers is assessed as the number of titers with the last plasma dilution at which the lysis or agglutination reaction of rabbit blood was observed [25]. However, the quantity of barn swallow plasma did not reach 50 μL in the majority of our samples, so we used 25 or 12.5 μL in the second or third titer depending on availability. Since the lysis reaction occurred in the first titers and we did not have plasma in some of them, we counted until the last titer in which the plasma had any activity (i.e., at the end of the agglutination) to avoid bias due to the absence of plasma in the first titers. These values were used as indicators of immunocompetence. The agglutination and lysis variables (in those samples for which we have both data, *N* = 16), were strongly positively correlated (*F* = 22.80, d.f. = 1, 14, *R*^2^ = 0.59, *P* = 0.0003, estimate (SE) = 2.13 (1.09)). Samples in which the activity was not detected were discarded from the analysis because we could not distinguish between no activity or if it occurred in the first titers. We obtained information for 49 individuals.

### Statistical analysis

The relationship among the antimicrobial activity of blood plasma and the background radiation level was first evaluated separately for each indicator bacterium through multiple regression analysis, in which radiation was considered the predictor variable, and inhibition capacity (i.e., halos size) the response variable. The false-discovery-rate (FDR) correction to establish the appropriate *Q* values was used (procedure of Benjamini and Hochberg), which were the calculated *P* values after FDR correction [33, 34].

To analyse the effect of background radiation on the global capacity of individuals of inhibiting bacterial growth, an antagonistic index was calculated as the average intensity of activity against the number of indicator bacteria tested for each sample (i.e., a sum of all halos sizes divided for the number of indicator bacteria tested for each sample). This index was log_10_-transformed to adjust to a normal distribution, and the variance was homogenous (Levene’s test, *F* = 0.67, d.f. = 1, 83, *P* = 0.41). In addition, all samples for which we had the values of antimicrobial activity against all the indicator strains (*N* = 70) were included in a Principal Component Analysis (PCA), to reduce the number of dependent variables and to assure statistical independence. PCA factors were rotated (varimax normalized), and their significance established by cross-validation. Antagonistic activity was summarized in five Principal Components (PC) that explained 75.47% of the total variance, with each PC explaining 29.13, 18.94, 10.16, 9.72 and 7.50%, respectively. The resulting PCA appropriately captured variation in the 12 variables since the estimated power of each of them varied between 0.68 and 0.89 [35]. The natural antibody and complement variable was log_10_-transformed to adjust to a normal distribution.

General Linear Models (GLM) were performed. On the one hand, ANCOVA was used in which background radiation and the other variables that could influence the immune responses (i.e., sex and age) were included as predictor variables, while the antagonistic index and the non-specific immune response mediated by natural antibody and complements were the dependent variables of the models. On the other hand, we used the MANCOVA analysis with the same dependent variables but with all factors obtained in the PCA. All analyses were performed with Statistica 7.1 software [36].

## Results

### Antimicrobial activity of plasma

Considering each indicator bacterium separately and after FDR correction, only *B*. *megaterium*, *L*. *monocytogenes* and *L*. *plantarum* had antimicrobial capacity in barn swallows that was not related to background radiation (Table 1). For the two latter species, results are marginally non-significant (*P* = 0.06, Table 1). In seven of the nine species for which a significant association was detected, activity against indicator bacteria was higher in individuals that were captured in more contaminated areas (Table 1). Thas was also the case when considering responses of the bacterial species that were marginally statistically significant (Table 1).

**Fig 2:**
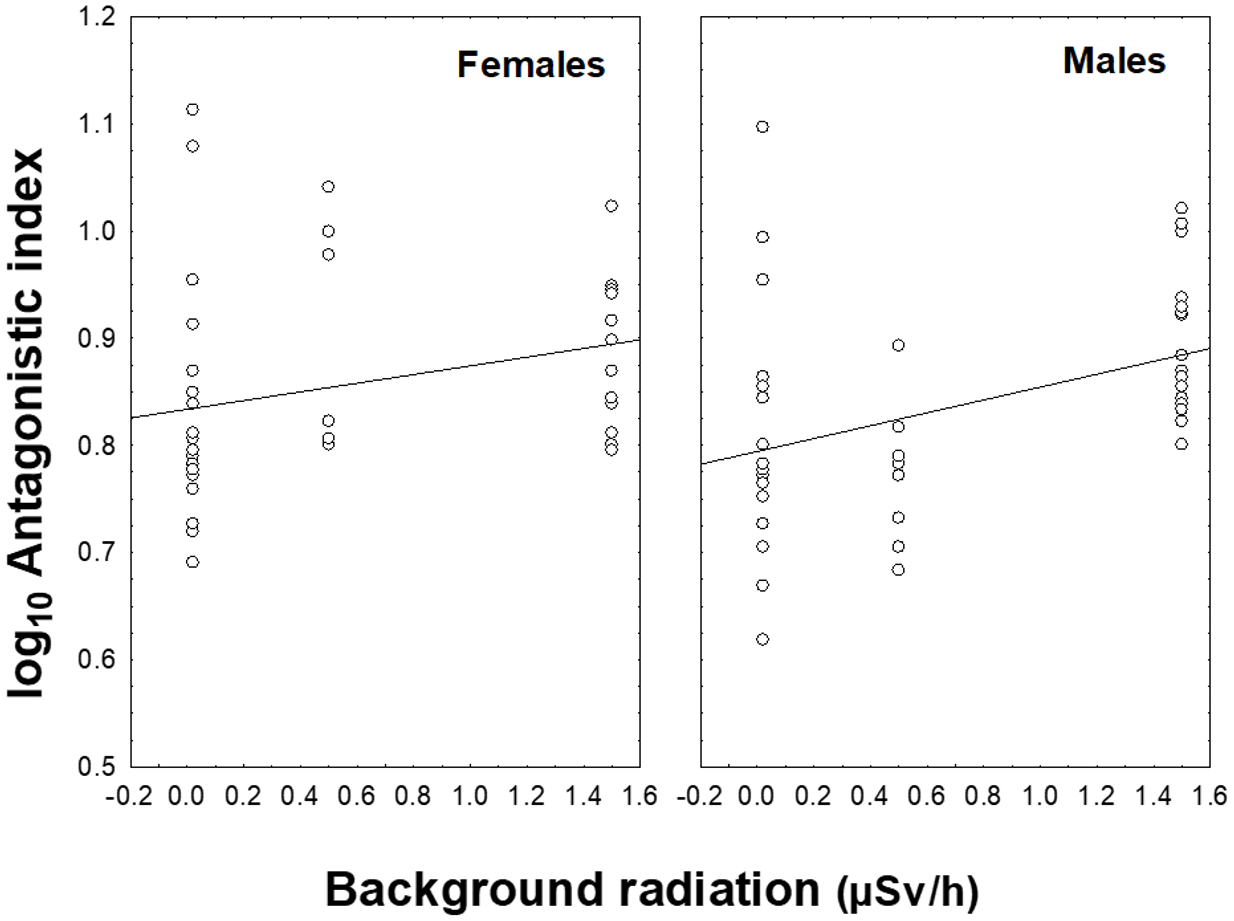
Variation in the global antagonistic index of female (left) and male barn swallows (right) under different background radiation levels.

The global antagonistic index was positively related to background radiation level (*F* = 5.22, d.f. = 1, 79, *P* = 0.02, β (SE) = 0.69 (0.30)), and neither sex (*F* = 2.07, d.f. = 1, 79, *P* = 0.15, β (SE) = 0.20 (0.14)) nor age (*F* = 2.65, d.f. = 1, 79, *P* = 0.11, β (SE) = 0.10 (0.22)), nor their interaction with radiation (*F* = 0.55, d.f. = 1, 79, *P* = 0.46, β (SE) = -0.11 (0.14) and *F* = 1.58, d.f. = 1, 79, *P* = 0.21, β (SE) = -0.41 (0.32) respectively), explained an additional significant proportion of variance (Fig 2).

Univariate analysis showed that the antagonistic index was reversed between sexes in *B*. *megaterium* (females were more resistant, *F* = 6.59, d.f. = 1, 65, *P* = 0.012), and *L*. *monocytogenes* (the opposite trend, *F* = 6.07, d.f. = 1, 82, *P* = 0.015). In the case of *E*. *faecium*, there was a marginally significant difference, with males being more resistant (*F* = 3.36, d.f. = 1, 83, *P* = 0.070).

We also calculated the percentage of strains that were inhibited per sample, in those that were tested at least against 6 strains (*N* = 75), but we did not find any effect of radiation (*F* = 0.06, d.f. = 1, 69, *P* = 0.80), sex (*F* = 1.92, d.f. = 1, 69, *P* = 0.17), nor age (*F* = 0.72, d.f. = 2, 69, *P* = 0.49).

When considering PCA values to characterize antimicrobial activity, we detected significant, positive effects of radiation level and sex, but no effect of age was found (Table 2). Moreover, the interaction between sex and radiation was marginally non-significant, while that was not the case for radiation and age (Table 2).

**Table 2:** Results of a multivariate general linear model that explored the effects of background radiation, sex and age on the first principal component of a PCA that included all antagonistic tests for bacteria.

Variable	Wilks’ λ	*F*_5,60_	*P*
**Radiation**	0.50	12.03	<0.0001
**Sex**	0.76	3.72	0.005
**Age**	0.97	0.34	0.88
Radiation*Sex	0.84	2.23	0.06
Radiation*Age	0.88	1.56	0.18

### Immunocompetence

Radiation and sex did not explain significant variation in the immune response (*F* = 0.96, d.f. = 1, 43, *P* = 0.33, β (SE) = 0.42 (0.43) and *F* = 0.13, d.f. = 1, 43, *P* = 0.72, β (SE) = 0.07 (0.19) respectively). However, there was a weak marginally non-significant association with age (*F* = 3.47, d.f. = 1, 43, *P* = 0.07, β (SE) = 0.36 (0.19)). None of the interactions were significant (radiation*sex: *F* = 0.23, d.f. = 1, 43, *P* = 0.63, β (SE) = 0.09 (0.19); radiation*age: *F* = 1.64, d.f. = 1, 43, *P* = 0.21, β (SE) = -0.59 (0.46)).

## Discussion

Our main finding was a positive relationship between capacity of barn swallow blood plasma to inhibit bacterial growth and ambient background radiation levels of breeding sites. Such a positive association was detected for more than 90% of the indicator bacteria assayed. Laboratory experiments exposing animals to ionising radiation have shown profound positive effects on susceptibility to a range of bacterial pathogens, especially *Listeria monocytogenes* [37, 38]. Interestingly, our results suggest the opposite pattern in natural populations, and here we discuss the possibility that the detected patterns were due to natural selection purging immune deficient individuals more rapidly in highly contaminated areas.

Interestingly, background radiation was the unique variable explaining variation in the global antagonistic index (which include the number of bacteria that one sample can inhibit, as well as the intensity of inhibition). We have previously demonstrated that birds from heavily radioactively contaminated areas were those more resistant to feather degradation by keratinolytic bacteria [28]. Microbial communities in Chernobyl are altered and the phenotypic condition of hosts has deteriorated as a consequence of the nuclear accident (see Introduction). Therefore, selection exerted by microorganisms on barn swallows may have purged those individual birds with low capacity to defend themselves from bacterial infections. Data on recaptures in subsequent years will allow us to assess whether there is a relationship between antimicrobial defence capacity and survival.

When considering the PCA results that summarise all antagonistic tests, background radiation again explained variability among individuals, but in this case there was also an effect of sex that differed among areas, i.e., plasma of males and females have different patterns of capacity to inhibit different bacterial species depending on the radiation level. Therefore, although both sexes of barn swallows captured in contaminated zones have a higher overall capacity to inhibit bacteria, they inhibit diverse bacterial taxa differentially. Males and females may encounter different bacterial communities since their behaviour differs during the breeding season, when we collected the samples. Although individuals of the two sexes collaborate in nest building, females spend more time inside the nests (laying eggs and incubating them) than males [26]. In feathers, bacterial communities differ among the sexes [11], and also in some defences against keratinolytic degradation [28]. In addition, the investment in reproduction may be lower in contaminated than in clean areas, since barn swallows experience reduced reproductive success in Chernobyl [26]. This difference is more evident for females that may invest more in self-maintenance.

Previous studies have detected reduced spleen size and lower lymphocyte counts and immunoglobulin concentrations in barn swallows breeding in Chernobyl in comparison with those breeding in non-contaminated control areas [19]. Moreover, individuals from Chernobyl were under higher immunological stress as showed by their heterophil:lymphocyte ratios [19]. Thus, previous studies have suggested a negative relationship between immunity and radiation. However, other variables indirectly related to immune capacity such as plasma antioxidant capacity did not vary with radiation level [39]. Our results also failed to detect the expected negative association between radiation and level of constitutive innate immune response. Previous studies of natural antibodies and complement in barn swallows breeding in uncontaminated zones showed that variation was explained by time during the breeding season and age of individuals [40]. In addition, they explained vital rates in females [40]. We also found a weak relationship with age in our samples, although other factors related to contamination may be influencing the results.

Although selection acting on the immune system of birds at Chernobyl during the last 30 years may explain inconsistency of results and the absence of a relationship between different levels of radioactivity and innate immune response of individuals affected by the Chernobyl catastrophe, this could also be due to different aspects of immune responses being investigated in different studies. Different immune components are not necessarily positively related to each other [41]. Although negative associations are consistent with a trade-off between different components of immunity [42], other studies detected positive or no associations among different lines of immunity [43]. Moreover, immunity is also related to a variety of physiological characteristics such as growth, reproduction and survival that depend on environmental factors and seasonality [44], which may have changed during the last 30 years.

In antagonistic tests we measured the specific response of plasma to bacterial growth, which may provide positive relationships between inhibition capacity and radiation. Therefore, it is possible that individuals that were able to defend themselves against bacterial infections are those that were favoured by natural selection. Interaction between radiation exposure and virulence changes among pathogen species and depends on the dynamic relationship between host and pathogens (reviewed in [3]). Considering possible changes driven by levels of radioactivity, both in the pathogens (e.g., biology, ecology and occurrence, which could ultimately increase their virulence) and in hosts (e.g., damage to the immune system), it is possible that the associated strong selection pressure on hosts may result in rapid adaptive evolution of the immune responses in birds living in this extreme and stressful environment. It is well documented that the Chernobyl disaster increased the occurrence of infectious diseases in humans [45, 46]. The opposite pattern was however detected when studying wild animal populations as shown by lower occurrence of infections caused by *Mycobacterium* spp. (which provokes tuberculosis) in animals living in contaminated zones than in animals living in Ukrainian control areas [47, 48], perhaps because of selective mortality.

Pathogenicity of bacteria is controversial, since the same species may live in some organisms without damaging them, while causing an infection under certain circumstances (e.g. [49]). In the species tested in our study, we could group *L*. *monocytogenes* [50], *E*. *faecalis* [51], *Proteus* sp. [52] and *Staphylococcus aureus* [53] as potentially pathogenic, but also the three *Bacillus* species since they are keratinolytic and could digest the feathers of their barn swallow hosts (e.g. [54]). However, the only species in which no relationship with radiation was found was *B*. *megaterium*. It is possible that for inhibiting feather-degrading microorganisms, other mechanisms such as uropygial secretions or physical resistance to degradation may be more developed [28]. Interestingly, both negative relationships between radiation and inhibition capacity were against *L*. *inocua* and *L*. *paracasei*, both widely accepted as being non-pathogenic bacterial species (e.g. [55, 56].

The mortality rate in birds living in Chernobyl is known to be much higher than that of conspecifics living in control populations [57]. If mortality was associated with immune capacity in animals, evolution of inmune response in the Chernobyl population should be detectable after a few years, assuming some amount of heritable genetic variation for immune response. Because microorganisms are able to rapidly evolve adaptive responses to radiation, animals that were unable to overcome immune depression caused by radiation will be purged from contaminated areas resulting in increased frequencies of adapted phenotypes in the population. The abundance of birds in highly contaminated areas of Chernobyl is about two thirds lower than in control areas [58]. Our results suggest that this elevated selection pressure has resulted in more immuno-competent populations, at least when considering adaptive immune responses mediated by particular antimicrobial agents (i.e. antibodies or immunoglobulins).

Infectious diseases are ubiquitous components of the biology of animals. Environmental pollution has the potential to change ecological interactions between species, including those among hosts and pathogens [59, 60, 61]. Although avian host-parasite relationships have been intensively investigated in wild bird populations from different points of view (e.g. [62, 63, 64]), studies of the effects of environmental pollution on these interactions are rare [61]. However, it is apparent that exposure of organisms to ionizing radiation has considerable effects on host-pathogen dynamics. In the present study, we add evidence to this by showing effects of background radiation, under field conditions, to the capacity to fight against bacterial infections. Therefore, infectious diseases are clearly an important factor influencing the health of wildlife exposed to elevated levels of ionizing radiation.
